# Coronary Microvascular Dysfunction Years After Cessation of Anabolic Androgenic Steroid Use

**DOI:** 10.1001/jamanetworkopen.2024.51013

**Published:** 2024-12-16

**Authors:** Yeliz Bulut, Jon Jarløv Rasmussen, Niels Brandt-Jacobsen, Jan Frystyk, Mario Thevis, Morten Schou, Finn Gustafsson, Philip Hasbak, Caroline Kistorp

**Affiliations:** 1 Department of Nephrology and Endocrinology, Copenhagen University Hospital-Rigshospitalet, Copenhagen, Denmark; 2 Department of Clinical Medicine, Faculty of Health and Medical Sciences, University of Denmark, Copenhagen, Denmark; 3 Department of Clinical Physiology and Nuclear Medicine, and Cluster for Molecular Imaging, Copenhagen University Hospital-Rigshospitalet, Copenhagen, Denmark; 4 Department of Endocrinology, Odense University Hospital, Odense, Denmark; 5 Faculty of Health Sciences, University of Southern Denmark, Odense, Denmark; 6 Center for Preventive Doping Research, German Sport University, Cologne, Germany; 7 Department of Cardiology, Herlev/Gentofte University Hospital, Herlev, Denmark; 8 Department of Cardiology, Copenhagen University Hospital-Rigshospitalet, Copenhagen, Denmark

## Abstract

**Importance:**

Long-term use of anabolic androgenic steroids (AASs) is associated with a high risk of left ventricular hypertrophy, heart failure with reduced systolic function, and early sudden death, with the mechanism of progression being understudied. Early and persistent impaired myocardial microcirculation could be of clinical importance and a potential underlying mechanism of frequent and early cardiac disease among individuals with AAS use and a future potential target for intervention.

**Objective:**

To investigate coronary microcirculation by measuring myocardial flow reserve (MFR) in men with current and former AAS use compared with controls with no prior AAS use, using cardiac rubidium 82 (^82^Rb) positron emission tomography/computed tomography (PET/CT).

**Design, Setting, and Participants:**

This cross-sectional study included men involved in recreational strength training without established cardiovascular disease grouped according to their history of AAS use. The study was conducted November 24, 2021, to August 16, 2023.

**Exposure:**

Cardiac PET/CT with ^82^Rb was performed at rest and after adenosine-induced stress.

**Main Outcome and Measure:**

The primary outcome of this study was the MFR among the study groups; a secondary outcome was the coronary calcium score. By definition, impaired myocardial microcirculation was determined using a cutoff of MFR less than 2, and subclinically impaired microcirculation was determined using a cutoff of MFR less than 2.5.

**Results:**

A total of 90 men (32 with current AAS use, 31 with former AAS use, and 27 controls) were included. Mean (SD) age was 35.1 (8.7) years. Elapsed duration since AAS cessation was a geometric mean of 1.5 (95% CI, 0.9-2.5) years. Eighteen men (58.1%) with former use discontinued AAS use more than 1 year before enrollment. Impaired MFR was present in those with current (6 [18.8%]) and former (1 [3.2%]) use, whereas no impairment was observed among the controls (*P* = .02). Subclinically impaired MFR was higher among men with current (9 [28.1%]) and former (8 [25.8%]) AAS use than the controls (1 [3.7%]) (*P* = .02). In a multivariable logistic regression model among men with former AAS use, every doubling of the accumulated weekly duration of AAS use (log_2_) was independently associated with a factor 2 increase in the risk of impaired MFR less than 2.5 (odds ratio, 2.1; 95% CI, 1.03-4.35; *P* = .04).

**Conclusions and Relevance:**

In this study, men with former AAS use displayed impaired MFR years after AAS cessation, suggesting impaired coronary microcirculation persists beyond active use.

## Key Points

**Question:**

Is the use of supraphysiologic doses of anabolic androgenic steroids (AASs) associated with persistent coronary microvascular dysfunction?

**Findings:**

In this cross-sectional study including 90 men, the prevalence of impaired myocardial flow reserve was increased among both those with current and former AAS use compared with controls. Among individuals with former AAS use, the accumulated duration of AAS use was independently associated with an increased risk of reduced myocardial flow reserve.

**Meaning:**

The findings of this study suggest that AAS use may be associated with impaired myocardial flow reserve, with possible persistent coronary microvascular dysfunction years after AAS cessation.

## Teaser

This cross-sectional study examines coronary microvascular status in younger men a year or more after cessation of the use of anabolic androgenic steroids.

## Introduction

The use of supraphysiologic anabolic androgenic steroids (AASs), including testosterone esters and its other synthetic derivatives, has become a growing public health concern worldwide for several years.^1^ With the increasing focus in younger men on a more muscular appearance, the use of AASs has become more widespread in that population.^2^ In a recent register-based study, men who were sanctioned for AAS use displayed a 2-fold increased risk of death from natural causes, the most frequent causes being cancer and cardiovascular disease (CVD).^3^ However, use of AASs may also be associated with other risk-taking propensities and use of other substances, which can contribute to the observed increased mortality. Cardiovascular death is especially of note, since AAS use has been associated with left ventricular (LV) hypertrophy and LV systolic and diastolic dysfunction in the absence of major epicardial atherosclerosis.^4,5^ Furthermore, these cardiac changes seem to persist, as individuals with former AAS use display decreased LV systolic function measured as impaired LV global longitudinal strain, a more sensitive marker of systolic function, years after AAS use.^4^ However, the underlying mechanism of cardiomyopathy with AAS use is understudied.

Coronary microvascular dysfunction refers to a spectrum of conditions with impacts in the structure and function of the myocardial microcirculation. Peak hyperemic myocardial blood flow (MBF) and myocardial flow reserve (MFR), the ratio between stress and rest MBF, quantified by rubidium-82 (^82^Rb) positron emission tomography/computed tomography (PET/CT) is the reference standard in the assessment of coronary microvascular function in patients without epicardial disease, where reduced MFR is well established as a possible indicator of future CVD.^6,7,8,9^ In experimental studies, prolonged AAS administration induces changes in oxidative biomarkers, and thus, oxidative stress, and vascular endothelial dysfunction.^10^ Furthermore, a general finding in younger individuals with AAS use is dyslipidemia and hypertension, which play a central role in vascular dysfunction.^11^ Therefore, we hypothesized that AAS use is associated with coronary microvascular dysfunction. The objective of the present study was to investigate myocardial microcirculation by MFR, using ^82^Rb PET/CT, in men with current and former AAS use compared with controls who had never used AASs.

## Methods

### Study Design and Procedures

The study was conducted in accordance with the Declaration of Helsinki^12^ and all relevant regulations in Denmark. The Danish Data Protection Agency approved the use of personal data, and ethical approval was granted by the Capital Regional Committee on Health Research Ethics. Oral and written informed consent were obtained from each participant before inclusion; participants did not receive financial compensation. In the reporting of this study, we followed the guidelines of the Strengthening the Reporting of Observational Studies in Epidemiology (STROBE) reporting guideline.^13^

We conducted a cross-sectional study including men aged 18 years or older involved in recreational strength training divided into 3 groups according to their self-reported AAS history: former AAS use, with a minimum of 4 weeks of AAS use, who had discontinued AAS intake a minimum of 3 months before the inclusion; current use; and controls, who had never used AASs or other performance-enhancing drugs. The participants were enrolled from November 24, 2021, to August 16, 2023. The primary outcome was MFR determined by ^82^Rb PET/CT among the 3 study groups, with the coronary calcium score as a secondary outcome.

We evaluated laboratory parameters known to be influenced by exogenous AAS use to confirm AAS status.^14^ The absence of current or recent use of AAS and the confirmation of ongoing use were confirmed using chromatographic-mass spectrometric tests on urine samples with analyses similar to commonly used routine controls.^15,16^ The urine samples were anonymized, and the urine analyses were performed blinded to group allocation at the Center for Preventive Doping Research at the German Sport University, Cologne, Germany.

Participants were recruited from gyms, social media platforms, word-of-mouth recruitment, or endocrine outpatient clinics of the Greater Copenhagen area, and participants were enrolled consecutively after assessment of fulfillments of inclusion and exclusion criteria on a first-come first-served basis. Participants who were enrolled from outpatient clinics were typically referred due to AAS-induced hypogonadism.

We aimed at matching the predefined study groups by age to be within the same decade and did not include criteria regarding the extent of recreational training, which did not seem feasible considering the high amount of training observed among participants with ongoing AAS use. Any history or signs of CVD was evaluated from personal interviews and by reviewing electronic hospital records. None of the participants reported angina or other cardiac-related symptoms before inclusion. Impaired microcirculation was defined as MFR less than 2.0 and subclinically impaired microcirculation was defined as MFR less than 2.5 based on a previous study among participants without manifest CVD.^7^

### ^82^Rb Cardiac PET/CT Scan

Participants underwent rest and adenosine stress cardiac PET/CT with ^82^Rb, using a 128-slice system (Siemens Biograph mCT PET system, Siemens Medical Solutions) using targeted injection doses of 1100 MBq ^82^Rb (Bracco Diagnostics Inc) intravenously with a constant flow rate of 50 mL/min. Adenosine was administered at a rate of 140 μg/kg/min over 6 minutes to induce maximum myocardial hyperemia. PET emission list-mode 3-dimensional data acquisition was started 2.5 minutes into the adenosine infusion. Both rest and stress scans continued for 6 minutes. The 6-minute PET acquisitions were reconstructed into dynamic image series consisting of 18 frames (1 × 10, 8 × 5, 3 × 10, 2 × 20, and 4 × 60 seconds), using the vendor iterative ordered-subset expectation-maximization 3-dimensional reconstruction method (2 iterations, 21 subsets), with corrections for time of flight and point-spread function. All data were smoothed using 6.5-mm gaussian postfiltering. Both rest and stress PET acquisitions were reconstructed into 8 cardiac phases (8 electrocardiogram gates), using data obtained between 150 and 360 seconds into the PET acquisition. Myocardial blood flow, in millimeters per gram per minute, was calculated using the Lortie model,^17^ and MFR was calculated as the ratio of the stress and rest MBF^18^ in dedicated software (QPET; Cedars-Sinai Medical Center).^19^ A low-dose CT for attenuation-correction purposes was acquired using a free breathing protocol, and a noncontrast breath-hold CT for coronary artery calcium score estimation was acquired. Left ventricular ejection fraction (LVEF) reserve was calculated as stress LVEF minus rest LVEF.

The participants were instructed not to consume caffeine drinks and food within 12 hours before the examination, and nitrates, phosphodiesterase-5-inhibitors, and dipyridamole-containing medications were discontinued at least 24 hours before the examination.

### Statistical Analysis

The sample size was based on a power calculation. The primary purpose of this study was to investigate MFR among the 3 study groups, and we considered a difference in myocardial blood flow of 0.5 mL/g/min clinically relevant. With an SD of measurement of global MFR by ^82^Rb of 0.5, based on a recent study in the department,^20^ a sample size of 30 participants was required in each of the 3 groups with a power of 90% and a reduced 2-sided α level of 0.015 (3 study groups and multiple comparisons in the study) to detect a difference of 0.5 mL/g/min. Due to a diagnosis of congenital heart disease (1 participant) and the withdrawal of informed consent (2 participants), we included 27 participants in the control group.

Normal distribution was evaluated by linearity in quantile plots and normal distributed variables are presented as mean (SD) and were compared, using analyses of variance with Tukey-Kramer post hoc adjustment for multiple comparisons. Nonnormally distributed variables were logarithmically transformed and are presented as geometric means (95% CIs). The variables are presented as medians (IQR) if a normal distribution was not achieved using logarithmic transformations, and the 3 groups were compared using the Kruskal-Wallis test. Categorical variables were compared using the χ^2^ test or Fisher exact test as appropriate, with post hoc Bonferroni adjustment for multiple comparisons. The nonparametric Cochran-Armitage test was used to assess trends of reduced MFR less than 2.5 and 2.0 across the study groups.

We used multivariable logistic regression to assess potential determinants of reduced MFR less than 2.5 among participants with former AAS use including the following covariates: age, systolic blood pressure, accumulated duration of AAS use, and time since AAS discontinuation. We considered *P* < .05 (2-tailed, unpaired) as the statistically significant threshold.

All analyses were performed using SAS Enterprise, version 9.4 (SAS Enterprise LLC) and Prism, version 10 (Dotmatics).

## Results

We included 90 men (32 with current AAS use, 31 with former AAS use, and 27 with nonuse [controls]) (Table 1). Overall mean (SD) age was 35.1 (8.7) years: 35.3 (8.7) years in those with former use and 31.2 (7.7) years among the controls (*P* = .15); individuals with current use were older than the controls (38.3 [8.4] years; *P* = .005).

**Table 1.** Baseline Characteristics and Cardiac ^82^Rb PET/CT Scan Data

Variable	No. (%)			*P* value^a^
	Controls	Current AAS use	Former AAS use	
Total participants	27 (30)	32 (36)	31 (34)	NA
Age, mean (SD), y	31.2 (7.7)	38.3 (8.4)	35.3 (8.7)	.007^b^
Participant recruitment				
Endocrine outpatient clinic	0 (0)	3 (9)	10 (32)	.002
Community	27 (100)	29 (91)	21 (68)	
Office-measured BP, mean (SD), mm Hg				
Systolic	123.2 (9.1)	142.3 (18.5)	133.8 (15.6)	<.00^c^
Diastolic	76.5 (7.9)	82.6 (11.4)	80.4 (9.3)	.05
Heart rate, mean (SD), beats/min	62.5 (9.0)	73.3 (12.9)	63.1 (12.9)	<.001^d^
Cardiac ^82^Rb PET/CT, mean (SD)				
LVEF rest, %	61 (5)	53 (7)	61 (6)	<.001^d^
LVEF stress, %	68 (5)	58 (8)	67 (5)	<.001^b^
LVEF reserve, %	7.8 (4.1)	4.8 (3.6)	6.8 (3.9)	.01^b^
LV mass rest, g	166 (23)	182 (20)	175 (22)	.03^b^
LV mass stress, g	180 (15)	194 (17)	183 (17)	.004^d^
LV mass (rest)/BSA, mean (SD), g/m^2^	81 (6)	84 (8)	81 (11)	.37
Coronary artery calcium score, median (range)	0 (0-1)	0 (0-16)	0 (0-42)	.32
Myocardial blood flow rest, mean (SD), mL/g/min	0.8 (0.2)	0.7 (0.2)	1.0 (0.3)	<.001^e^
Myocardial blood flow stress, mean (SD), mL/g/min	2.9 (0.5)	2.4 (1.0)	3.1 (0.6)	<.001^d^
Myocardial flow reserve, mean (SD)	3.7 (0.8)	3.3 (1.2)	3.3 (0.9)	.15
Myocardial flow reserve <2.5	1 (3.7)	9 (28.1)	8 (25.8)	.02^c^
Myocardial flow reserve <2.0	0	6 (18.8)	1 (3.2)	.02^b^
Laboratory analyses				
Hematocrit (blood), mean (SD), %	42 (2)	50 (3)	44 (3)	<.001^f^
LDL-C (plasma), mean (SD), mg/dL	92.7 (23.2)	127.4 (42.3)	112.0 (38.6)	.002^b^
Total testosterone (serum), ng/dL, median (IQR)	489.1 (403.5-576.4)	>1152.7 (489.9->1152.7)	374.6 (317.0-461.1)	<.001^f^
Training and history of AAS use				
Recreational strength training, mean (SD), h/wk	7 (4)	8 (4)	6 (3)	.09
Accumulated duration of AAS use, geometric mean (95% CI), wk	NA	213 (138-328)	110 (74-163)	.03
Duration since AAS cessation, geometric mean (95% CI), y	NA	NA	1.5 (0.9-2.5)	NA
Duration since AAS cessation >12 mo	NA	NA	18 (58.1)	NA
Body composition				
Lean mass index, mean (SD), kg/m^2^	20 (2)	23 (2)	21 (2)	<.001^d^
BSA, mean (SD), m^2^	2.1 (0.1)	2.2 (0.1)	2.2 (0.2)	.15
Body fat, mean (SD), %	22 (4)	19 (3)	24 (4)	<.001^d^
Smoking				
Never	18 (67)	12 (38)	12 (39)	.09
Previously	2 (7)	10 (31)	7 (23)	
Currently	7 (26)	10 (31)	12 (39)	
Use of illicit drugs other than AAS				
Never	11 (41)	5 (16)	3 (10)	.005^e^
Previously	14 (52)	20 (63)	27 (87)	
Currently	2 (7)	7 (22)	1 (3)	

Abbreviations: ^82^Rb; rubidium 82; AAS, androgenic anabolic steroid; BP, blood pressure; BSA, body surface area; LDL-C, low-density lipoprotein cholesterol; LV, left ventricle; LVEF, left ventricle ejection fraction; NA, not applicable; PET/CT, positron emission tomography/computed tomography.

SI conversion factors: To convert hematocrit to proportion of 1.0, multiply by 0.01; LDL-C to millimoles per liter, multiply by 0.0259; testosterone to nanomoles per liter, multiply by 0.0347.

^a^ *P* values compared across the groups unless otherwise stated.

^b^ Controls differ from individuals with current AAS use.

^c^ Controls differ from the other 2 groups.

^d^ Participants with current AAS use differ from the other 2 groups.

^e^ Participants with former AAS use differ from the other 2 groups.

^f^ Participants with former AAS use differ from those with current AAS use.

The accumulated duration of AAS use was longer among individuals with current use (geometric mean, 213; 95% CI, 138-328 weeks) than former use (geometric mean, 110; 95% CI, 74-163 weeks) (*P* = .03). The elapsed time since AAS cessation among those with former use was a geometric mean of 1.5 (95% CI, 0.9-2.5) years, and 18 (58.1%) of those with former use had discontinued AAS use more than 1 year before study inclusion. In analysis of urine samples, AAS was detected in 3 participants (10%) categorized as having former use vs 29 (91%) categorized as having ongoing use (eTable in Supplement 1). Higher mean (SD) systolic blood pressure was noted in individuals with current (142.3 [18.5] mm Hg) and former (133.8 [15.6] mm Hg) AAS use than controls (123.2 [9.1] mm Hg) (*P* < .001). The levels of low-density lipoprotein cholesterol were higher among individuals with current use (mean [SD], 127.4 [42.3] mg/dL) than in those with former use (112.0 [38.6] mg/dL) and controls (92.7 [23.2] mg/dL) (*P* = .002). Individuals with current use (mean [SD], 19% [3%]) demonstrated lower body fat compared with controls (22% [4%]) and those with former (24% [4%]) AAS use (*P* < .001). A higher proportion of participants with former use (27 [87%]), compared with those with current use (20 [63%]) and the controls (14 (52%]) have previously used illicit drugs other than AASs (*P* = .005).

**Figure 1.**
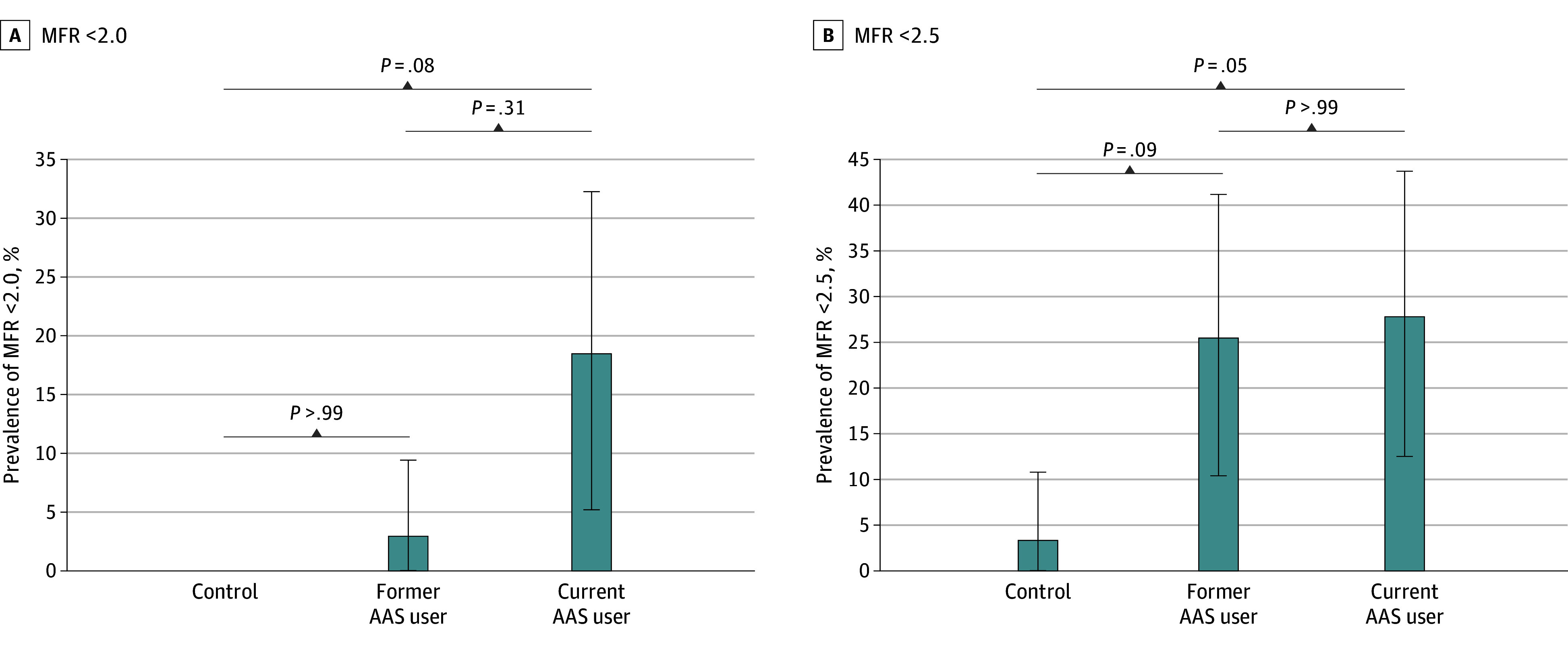
Prevalence of Impaired Myocardial Flow Reserve and Subclinically Impaired Myocardial Flow Reserve Prevalence of impaired MFR (<2.0; A) and subclinically impaired MFR (<2.5; B) among the 3 study groups. Error bars indicate 95% CIs. Overall *P* = .02 from Fisher exact test. Pairwise comparisons for *P* values shown. AAS indicates androgenic anabolic steroid; MFR, myocardial flow reserve.

Impaired MFR (<2.0) was present in participants with current (6 [18.8%]) and former (1 [3.2%]) AAS use, but not in the controls (overall *P* = .02) (Table 1, Figure 1A) (trend analysis, *P* = .005). Post hoc adjusted pairwise comparison between participants with current use and controls was not significant (*P* = .08). The prevalence of subclinically impaired MFR (<2.5) was higher in participants with current (9 [28.1%]) and former (8 [25.8%]) use than in the controls (1 [3.7%]) (overall *P* = .02) (Table 1, Figure 1B) (trend analysis, *P* = .03). Adjusted pairwise comparison between current users and controls (*P* = .05), and between individuals with former use and controls (*P* = .09) was not significant. None of the participants categorized as having former use but with detectable amounts of AAS in urine presented with MFR (<2.5).

Analyzed as a continuous variable, MFR values were numerically lower in participants with current (mean [SD], 3.3 [1.2] mL/g/min) and former (mean [SD], 3.3 [0.9] mL/g/min) use compared with controls (mean [SD], 3.7 [0.8] mL/g/min); however, the results were not statistically significant (*P* = .15) (Table 1). The coronary calcium score was within the reference range across all 3 study groups (Table 1).

We did not observe any association between selected variables, including cardiac function and structure, as LVEF and LV mass in univariable regression analyses on participants with former AAS use with MFR less than 2.5 as the dependent variable, since only accumulated weekly duration of AAS use was significant (B, 0.77; 95% CI, 0.10-1.43 weeks; *P* = .02) (Table 2).

**Table 2.** Univariable Regression Analyses of Former AAS Use With Myocardial Flow Reserve Less Than 2.5 as Dependent Variable

Variable	B (95% CI)	*P* value
Age (per 10 y)	0.57 (−0.34 to 1.47)	.22
Office systolic blood pressure (per 10 mm Hg)	−0.09 (−0.61 to 0.43)	.74
LVEF rest, %	0.01 (−0.13 to 0.14)	.94
LVEF stress, %	−0.03 (−0.17 to 0.13)	.77
LVEF reserve, %	−0.09 (−0.31 to 0.14)	.44
LV mass rest, g	0.02 (−0.02 to 0.06)	.37
LV mass (rest)/LBM, g/kg	0.07 (−2.25 to 2.38)	.96
Hematocrit (blood), %	−1.30 (−31.44 to 28.85)	.93
LDL-C (plasma), mg/dL	0.02 (−0.86 to 0.90)	.97
Lean mass index, kg/m^2^	0.21 (−0.23 to 0.65)	.35
Body fat, %	−0.07 (−0.32 to 0.18)	.58
Previous/current illicit drug use other than AAS	5.81 (−293 to 305)	.97
Smoking history: previous/current	0.03 (−0.79 to 0.86)	.94
Accumulated duration of AAS use (log_2_), wk	0.77 (0.10 to 1.43)	.02
Duration since AAS cessation (log_2_), y	0.08 (−0.30 to 0.45)	.69

Abbreviations: AAS, androgenic anabolic steroid; LBM, lean body mass; LDL-C, low-density lipoprotein cholesterol; LV, left ventricle; LVEF, left ventricular ejection fraction.

**Figure 2.**
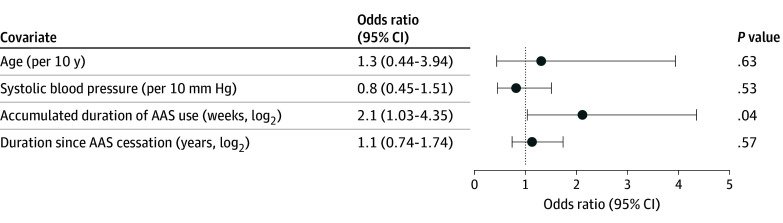
Multivariable Logistic Regression Analyses on 31 Individuals With Former Androgenic Anabolic Steroid (AAS) Use Using Myocardial Flow Reserve Less Than 2.5 as the Dependent Variable

In multivariable logistic regression analysis in participants with former use, every doubling of accumulated weekly duration of AAS use was independently associated with a factor 2 increase in the risk of subclinically impaired MFR (<2.5) (odds ratio, 2.1; 95% CI, 1.03-4.35; *P* = .04), while every doubling of elapsed duration since AAS cessation in years was not associated with MFR less than 2.5 (Figure 2).

## Discussion

The main finding of the present study is that impaired MFR was present among younger men with ongoing or previous use of AASs, suggesting supraphysiologic doses of testosterone and synthetic steroids could have adverse impacts in coronary microcirculation.

This study highlights reduced MFR as a potential contributing factor to the cardiac dysfunction observed in larger populations of individuals using AASs.^4,5^ Furthermore, the impaired myocardial microcirculation in individuals using AASs seems to persist despite cessation of AAS use. This is further substantiated by the independent association noted between longer accumulated duration of AAS use and increased risk of reduced MFR, suggesting a potential dose-response effect between AAS use and coronary microvascular dysfunction.

Several underlying pathophysiologic mechanisms might be considered. Supraphysiologic doses of AASs are suggested to induce endothelial dysfunction in younger males by inducing oxidative stress,^21^ and the increased sympathetic activity and aldosterone activation in individuals with AAS use could mediate coronary microvascular dysfunction by inducing vasoconstriction.^22^

Coronary endothelial dysfunction has been reported among patients with early asymptomatic heart failure and is hypothesized to result in recurrent myocardial ischemia or small myocardial infarcts,^6,23^ which further are suggestive of future progression of overt heart failure in individuals with and without known CVD.^24^ Coronary microvascular dysfunction may not only lead to increased mortality by increasing the risk of heart failure; studies in patients with cardiomyopathy have documented an increased risk of ventricular arrythmias in patients with reduced MFR,^25^ particularly if the MFR reduction is heterogeneous in the LV.^26^ This might explain unexpected cardiac death in individuals with current or prior AAS use even in the absence of overt heart failure.

Additionally, impaired MFR is independently suggestive of a future increased cardiac event rate among patients with symptoms of ischemia but without significant coronary artery lesions.^27^ In the present study, the association with MFR did not seem to be completely reversible.

Some clinical studies reported that AAS-induced cardiomyopathy could be reversible, since impaired systolic function as LVEF recovered following AAS discontinuation.^28^ In contrast, impaired LV strain seems to persist years after AAS discontinuation, even though LVEF was not affected.^4^

This apparent discrepancy may be further illuminated by the HAARLEM study, where no long-term impacts in short-term use of AASs as 1 cycle on cardiac systolic function and LVM was reported.^29^ These findings align with the notion of a dose-response effect between duration of AAS use and steroid-induced cardiomyopathy. Case report studies suggested that AAS-induced severe systolic heart failure might be reversible, but, in general, the recovery was after intensive heart failure treatment, while in some patients advanced heart failure was not reversible and the patients required heart transplant.^30,31^

In the TRAVERSE trial, testosterone replacement therapy among men aged 45 to 80 years with functional hypogonadism and established or at high risk for CVD did not find an increased risk of major adverse cardiac events compared with placebo, which was the primary outcome.^32^ However, the treatment increased the incidence of atrial fibrillation and pulmonary embolism. These results suggest that treatment using physiologic doses of testosterone replacement therapy may be safe for the cardiovascular system. However, testosterone and other synthetic AASs are usually administered at supraphysiologic doses among individuals using AASs, which seems to play a role in the development of changes in cardiac function.

Our findings suggest that a proportion of younger men who use AASs risk development of coronary microvascular dysfunction at an early age, and thus, potentially carry an increased risk of cardiac disease, even years after AAS use.

### Limitations

This study has limitations that need to be considered. The main outcome of the present study—group difference in mean MFR—on which the power calculation was based, was numerically greater, but not statistically significant between the groups. We noted an overall significant difference among the groups in impaired and subclinically impaired MFR, but the *P* values did not reach statistical significance in post hoc pairwise comparisons. This could be due to the fact that the sample size was not adequate to assess prevalences of impaired MFR with post hoc adjustment for multiple comparisons.

Group allocation of participants was based on self-reported AAS history. This has previously been shown to be a reliable method when supported by biomarkers known to be influenced by supraphysiologic doses of AASs.^14,33^ To substantiate our findings, we performed urinalyses in accordance with tests for banned performance-enhancing drugs to confirm the current absence of exogenous AASs among controls and participants reporting former use and those indicating ongoing AAS use. Furthermore, participants with former and current AAS use presented higher systolic blood pressure than the controls, and the levels of low-density lipoprotein cholesterol were higher among participants with current use than the controls. Individuals with current use showed lower body fat compared with the other groups, and use of illicit drugs other than AASs differed former users compared with the other groups. These differences in classical cardiovascular risk factors should be taken into account as potential confounders of the main findings of the present study. However, the data suggest no association with MFR less than 2.5 with these cardiovascular risk factors.

We cannot exclude selection bias. However, to minimize this risk, we included participants irrespective of duration and extent of AAS use. Epicardial coronary artery disease would affect MFR even in the absence of microvascular disease. However, the calcium score within the reference range in all participants suggests that coronary stenoses were highly unlikely.

The study included a relatively low number of participants, which may influence the results. In addition, we cannot exclude other unmeasured variables that may explain the observed association between AAS use and impaired MFR.

## Conclusions

In this cross-sectional study, both participants with current and former AAS use displayed impaired MFR, suggesting an association between AAS use and worse MFR. This association has the potential to impact coronary microcirculation following AAS use that may persist beyond cessation.

## Notes

Supplement 1. **eTable.** Anabolic Androgenic Steroids Detected in Urine Samples

Supplement 2.
Data Sharing Statement
