# Supplementary material for: Coronary Microvascular Dysfunction Years After Cessation of Anabolic Androgenic Steroid Use
Source: JAMA Netw Open. 2024 Dec 16;7(12):e2451013. doi: 10.1001/jamanetworkopen.2024.51013 (PMC11650407; doi:10.1001/jamanetworkopen.2024.51013)
Supplement: Supplement 1. — eTable. Anabolic Androgenic Steroids Detected in Urine Samples [file jamanetwopen-e2451013-s001.pdf]

Supplementary Online Content

Bulut Y, Rasmussen JJ, Brandt-Jacobsen N, et al. Coronary microvascular dysfunction years after cessation of anabolic androgenic steroid use. *JAMA Netw Open*. 2024;7(12):e2451013. doi:10.1001/jamanetworkopen.2024.51013

eTable. Anabolic Androgenic Steroids Detected in Urine Samples

This supplementary material has been provided by the authors to give readers additional information about their work.

© 2024 Bulut Y et al. *JAMA Network Open.*

eTable. Anabolic androgenic steroids detected in urine samples

Study groups<br>No. (%)	Urine test negative for AAS<br>No. (%)	Urine test positive for AAS<br>No. (%)	Urine test inconclusive for AAS*<br>No. (%)	Components of AAS<br>No. (%)
Current AAS users, 32 (36)	1 (3)	29 (91)	2 (6)	Testosterone, 26 (81)<br>Metenolone, 19 (59)<br>Drostanolone, 15 (47)<br>Mesterolone, 13 (41)<br>Nortestosterone, 9 (28)<br>Trenbolone, 8 (25)<br>Stanozolol, 5 (16)<br>Anastrozol, 5 (16)<br>Oxandrolone, 4 (13)<br>Oxymetholone, 3 (9)<br>Metandienone, 2 (6)<br>Methylclostebol, 2 (6)<br>Boldenone, 2 (6)<br>Clostebol, 1 (3)<br>Methyltestosterone, 1 (3)<br>Stenbolone, 1 (3)
Former AAS users, 31 (34)	27 (87)	3 (10)	1 (3)	Drostanolone, 2 (6)<br>Stanozolol, 1 (3)<br>Trace amounts of stanozolol, 1 (3)
Controls, 27 (30)	27 (100)	0	0	

*Inconclusive test due to insufficient urine volume

Abbreviation: AAS, androgenic anabolic steroids.

© 2024 Bulut Y et al. *JAMA Network Open.*
