# Supplementary material for: Coronary Microvascular Dysfunction Years After Cessation of Anabolic Androgenic Steroid Use
Source: JAMA Netw Open. 2024 Dec 16;7(12):e2451013. doi: 10.1001/jamanetworkopen.2024.51013 (PMC11650407; doi:10.1001/jamanetworkopen.2024.51013)
Supplement: Supplement 2. — Data Sharing Statement [file jamanetwopen-e2451013-s002.pdf]

Data Sharing Statement

Bulut. Coronary Microvascular Dysfunction Years After Cessation of Anabolic Androgenic Steroid Use. *JAMA Netw Open*. Published December 16, 2024.
doi:10.1001/jamanetworkopen.2024.51013

Data
Data available: No

Additional Information
Explanation for why data not available: Restrictions apply to the availability of all data generated to preserve patient confidentiality. The corresponding author will on request detail the restrictions and any conditions under which access to some data may be provided.
